# Prognostic impact of body composition in hepatocellular carcinoma patients with immunotherapy

**DOI:** 10.1080/07853890.2024.2395062

**Published:** 2024-08-27

**Authors:** Lilong Zhang, Xinyi Li, Kunpeng Wang, Min Wu, Wenhui Liu, Weixing Wang

**Affiliations:** a Department of General Surgery, Renmin Hospital of Wuhan University, Wuhan, China; b Hubei Key Laboratory of Digestive System Disease, Wuhan, China; c General Surgery Laboratory, Renmin Hospital of Wuhan University, Wuhan, China; d Department of Oncology, Third People’s Hospital of Honghe Prefecture, Gejiu, China; e Department of Dermatology, Liaocheng People’s Hospital, Liaocheng, China

**Keywords:** Body composition, sarcopenia, skeletal muscle index, subcutaneous adipose index, immune checkpoint inhibitors, hepatocellular carcinoma

## Abstract

**Objective:**

This study aims to examine the possible relationship between body composition parameters, sarcopenia, and clinical outcomes in hepatocellular carcinoma (HCC) patients who received immune checkpoint inhibitor (ICI) treatment.

**Methods:**

Three online databases, including Embase, PubMed, and the Cochrane Library, were thoroughly searched for literature describing the relationship between body composition parameters, sarcopenia, and outcomes of ICI-treated HCC patients from the start of each database to 21 January 2024. The Newcastle-Ottawa Scale was used to rate the quality of the studies. The assessed outcomes included hazard ratio (HR) for OS and PFS, as well as odds ratio (OR) for ORR and DCR.

**Results:**

This analysis included a total of 15 articles with a combined patient cohort of 1543 individuals. The results demonstrated that HCC patients with low skeletal muscle index (SMI) had significantly inferior OS (HR: 1.68, *p* < 0.001), PFS (HR: 1.45, *p* < 0.001), ORR (OR: 0.64, *p* = 0.044), and DCR (OR: 0.58, *p* = 0.009) compared to those with high SMI. The presence of sarcopenia in HCC patients was significantly related to poorer OS (HR: 1.63, *p* < 0.001) and PFS (HR: 1.48, *p* < 0.001), as well as a lower ORR (OR: 0.64, *p* = 0.020) and DCR (OR: 0.58, *p* = 0.007) in comparison to those without sarcopenia. Subgroup analysis demonstrated that these findings were consistent with the multivariate analysis. Moreover, high subcutaneous adipose index (SAI) levels were associated with better OS (HR: 0.46, *p* = 0.001) and PFS (HR: 0.68, *p* = 0.021) than those with low SAI levels.

**Conclusion:**

The presence of sarcopenia and low SMI in HCC patients undergoing treatment with ICIs was found to be related to inferior treatment response and reduced long-term effectiveness.

## 1. Introduction

Hepatocellular carcinoma (HCC) is the fourth-highest cause of cancer deaths worldwide [1]. Studies have confirmed that hepatitis B virus and hepatitis C virus infection, aflatoxin, drinking water pollution, alcohol, cirrhosis, sex hormones, nitrosamines, trace elements, non-alcoholic fatty liver disease, and so on are associated with HCC [2–6]. Significant advancements in HCC systemic therapy have been made in the previous 5 years [7]. In addition to multiple targeted therapy agents, immune checkpoint inhibitors (ICIs) have been authorized as advanced HCC treatment options, either alone or in combination [8]. These immunotherapies have the potential to boost anti-tumor activity and overcome immune tolerance mechanisms in the tumor microenvironment [9]. However, the objective response rate of ICI monotherapy ranges from 14 to 20% [10]. Researchers’ attention has been called to the hunt for new biomarkers for predicting treatment outcomes as a result of the low response rate. The identification of reliable positive and negative biomarkers would enable personalized treatment selection based on the anticipated effectiveness of therapy, thereby avoiding the costs associated with ineffective treatments.

Sarcopenia, characterized by the loss of skeletal muscle mass and function, has emerged as a significant aspect of cancer-related cachexia, capturing the attention of researchers due to its clinical implications [11]. In clinical practice, computed tomography (CT) is frequently employed to diagnose sarcopenia by assessing skeletal muscle mass, as CT scans are routinely conducted for tumor diagnosis and therapy planning and offer high specificity and accuracy in evaluating muscle distribution [12]. Recent investigations have identified sarcopenia as a prognostic indicator in HCC patients with surgery, radiotherapy, and chemotherapy [13,14]. However, the clinical influence of baseline body composition parameters or sarcopenia on the effectiveness of ICI therapy in HCC patients remains unclear. Although previous meta-analyses have explored the relationship between sarcopenia and ICI efficacy, all of these analyses were pan-cancer and included only 1–2 studies on HCC [15–17], and it was not possible to judge the role of body composition or sarcopenia in the treatment of ICI in patients with HCC.

This study aimed to investigate the relationship between pre-treatment body composition parameters, sarcopenia, and outcomes of ICI-treated HCC patients, such as treatment response and long-term survival.

## 2. Methods

### 2.1. Search strategy

Starting 21 January 2024, we performed a computerized search of bibliographic databases, including EMBASE, PubMed, and the Cochrane Library, using specific search terms, such as ‘immune checkpoint inhibitors’ [Mesh], ‘ICIs’, ‘PD-1 Inhibitors’, ‘PD-L1 Inhibitors’, ‘CTLA-4 Inhibitors’, ‘Pembrolizumab’, ‘Nivolumab’, ‘Atezolizumab’, ‘Ipilimumab’, ‘Camrelizumab’, ‘Sintilimab’, ‘Tislelizumab’, ‘Toripalimab’, ‘Envafolimab’, ‘Body Composition’ [Mesh], ‘Skeletal muscle index’, ‘SMI’, ‘Psoas muscle index’, ‘PMI’, ‘Sarcopenia’, ‘Skeletal muscle density’, ‘SMD’, ‘Myosteatosis’, ‘Visceral adipose index’, ‘VAI’, ‘Intramuscular adipose index’, ‘Total adipose index’, ‘TAI’, ‘Subcutaneous adipose index’, and ‘SAI’, in all fields. The search was limited to human studies published in English. It is worth noting that, to ensure the greatest possible chance of a complete search, we did not limit the cancer type at the time of the search. For further information, please refer to Supplementary Material 1 for the detailed search strategy. Furthermore, we also searched for grey literature on Google Scholar and manually examined the reference lists of eligible studies. As advised by the Cochrane collaboration, the search results from manual and electronic sources were integrated into Covidence software for data management.

### 2.2. Inclusion and exclusion criteria

We established the following inclusion criteria for selecting articles: (i) studies of HCC patients; (ii) treatment with ICIs; (iii) evaluation of the prognostic significance of baseline body composition parameters (before ICI therapy); and (iv) reporting of at least one of the following outcomes: overall survival (OS), progression-free survival (PFS), objective response rate (ORR), or disease control rate (DCR). The following were the exclusion requirements: (i) study designs, such as animal studies, reviews, case reports, and conference abstracts; and (ii) neither the text nor the published data were used to determine the hazard ratio (HR) or odds ratio (OR) for outcomes. When multiple studies had overlapping patient populations, we only selected articles with comprehensive data and strict methodology [18].

### 2.3. Data extraction and quality assessment

During data extraction, we collected essential information, including author, year, study region, study period, study design, demographical information (sample size, age, sex, and cancer type), treatment, outcomes, and body compositions (testing methods, site, analytical software, and cut‐point). HRs, ORs, and 95% confidence intervals (CIs) were extracted primarily from multivariate analyses; otherwise, from univariate analyses or using Engauge Digitizer to extract from survival analysis charts. The Newcastle-Ottawa Scale (NOS) score was used to evaluate the quality of observational studies. Studies with a score >6 were considered high-quality literature. We assigned nine points worth of quality-related criteria to the domains of patient selection, study comparability, and study endpoints. All of the above steps, including literature retrieval and screening, data extraction, and literature quality assessment, were performed and cross-checked independently by three researchers, and disputes were encountered and referred to the senior author.

### 2.4. Statistical methods

Stata 15.0 was used to perform the statistical analysis. Forest plots were used to visualize the results. Cochran’s *Q* test and *I*^2^ statistics were used to estimate heterogeneity. Significant heterogeneity was defined as a *p*-value of <0.1 and an *I*^2^ value >50%. In this case, a random-effect model with the DerSimonian-Laird was used; otherwise, a fixed-effect model with the Inverse Variance method was used. Publication bias was evaluated using Egger’s regression test [19] and Begg’s test [20]. Sensitivity analyses were conducted by removing each study to assess the robustness of the results. Subgroup analyses were performed regarding Cox regression analysis, body compositions, and methods of body composition analysis. The threshold for statistical significance was a two-tailed *p*-value <0.05.

## 3. Results

### 3.1. Search results and included studies

**Figure 1.**
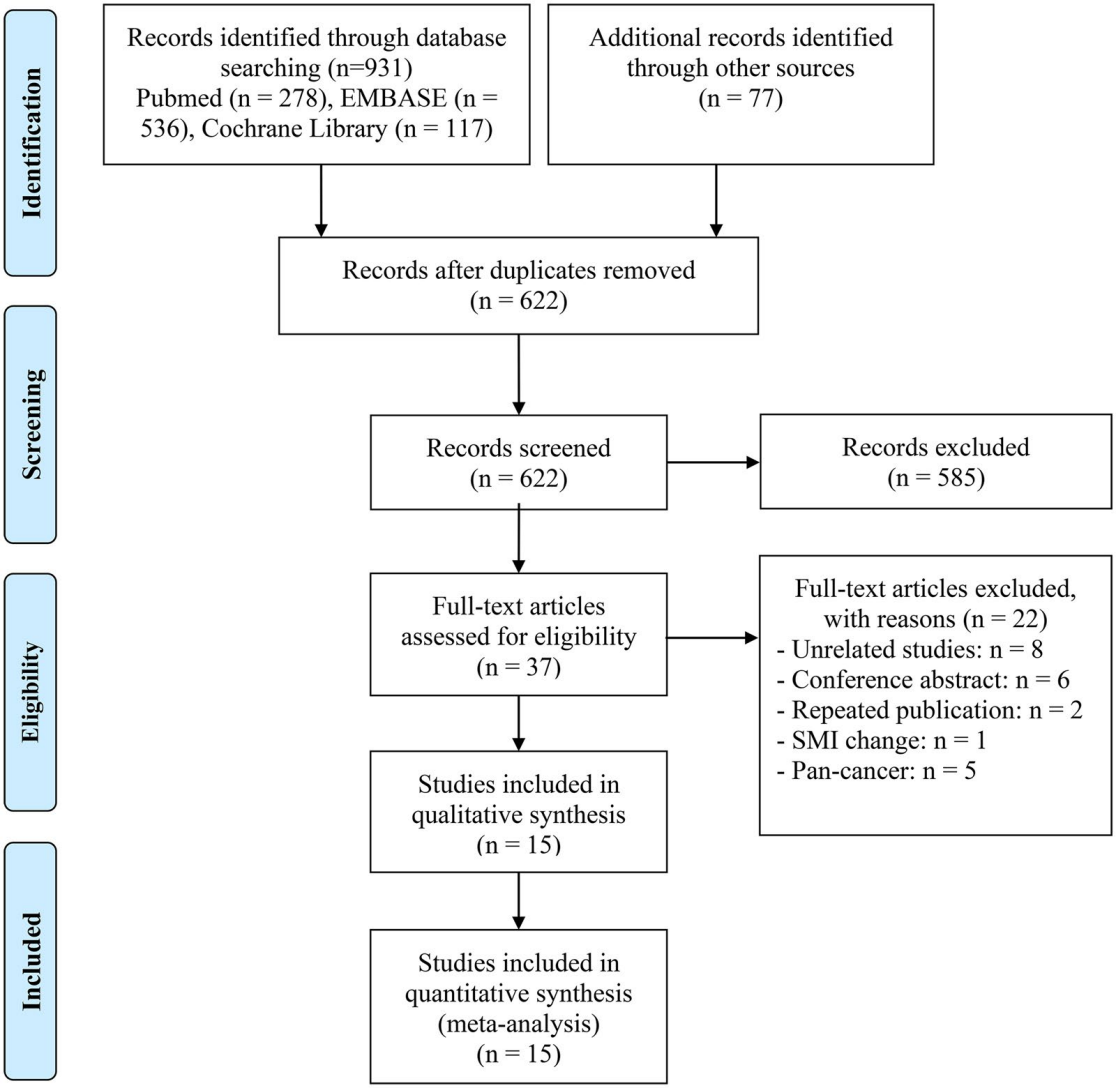
The flow diagram of identifying eligible studies.

Figure 1 illustrates the findings presented in the PRISMA flow diagram. A total of 1008 articles were identified through database searches and manual searches. After the removal of duplicates, 622 unique articles were identified. Upon evaluating the titles and abstracts, 585 articles were deemed ineligible and subsequently excluded. Thirty seven articles were reviewed in full‐text, and fifteen studies met the criteria for inclusion and were included in the analysis [21–35].

### 3.2. Study characteristics

The main characteristics of the studies included in this analysis are shown in Table 1. A total of 1543 patients with a mean or median age ranging from 51.2 to 80 years were included, and sample sizes ranged from 35 to 229 individuals. Nine studies were conducted in China; five were performed in Japan; and one was performed in Korea. Ten studies used CT, and three studies utilized CT or magnetic resonance images (MRI) to measure body composition in the third lumbar vertebrae, while two studies used a bioelectrical impedance assay (BIA) device to measure the above metrics. Thirteen studies used the skeletal muscle index (SMI) to diagnose sarcopenia, and two studies used the psoas muscle index (PMI) to define sarcopenia. Skeletal muscle density (SMD) was also used in two studies as a gauge of myosteatosis. All the included studies were retrospective. The thirteen papers received Newcastle-Ottawa Scale (NOS) scores from 6 to 8, indicating a minimal likelihood of bias (Table 1).

**Table 1.** Main characteristics of the studies included.

Study	Region	Period	Design	Sample size	Age	Male/female	Treatment	Body compositions and outcomes	Methods, site, and software	Cut‐point	NOS
Hiraoka et al. 2023	Japan	09/2020–12/2021	R	229	73 (70–79)^c^/74 (68–80)^c1^	186/43	Atezolizumab plus bevacizumab	SMI [OS(M), PFS(M), ORR(U), DCR(U)]	CT, L3, Synapse Vincent 3D image analysis system	≤42 cm^2^/m^2^ in males and ≤38 cm^2^/m^2^ in females	8
Zhang et al. 2023	China	02/2018–11/2021	R	56	59 (52–70)^c^	50/6	Camrelizumab, Pembrolizumab, Nivolumab, Tislelizumab, Sintilimab, Toripalimab	SAI [OS(U), PFS(U)]	CT, L3, ImageJ	Median value (39.27 cm^2^/m^2^)	7
Luo et al. 2023	China	11/2019–10/2021	R	124	54 (27–76)^b^	140/0	Sintilimab, Camrelizumab	PMI (OS)	CT/MRI, L3, SliceOmatic	≤5.5409 cm^2^/m^2^	7
Xiong et al. 2023	China	01/2019–01/2022	R	74	56 (35–79)^b^	63/11	Anti-PD-(L)1 antibody	SMI, SAI, VAI, TAI [OS(M), PFS(M)]	CT, L3, QCT Pro workstation	SMI, <43 cm^2^/m^2^ in male and <41 cm^2^/m^2^ in female in patients with a BMI < 25 kg/m^2^, <53 cm^2^/m^2^ in male and <41 cm^2^/m^2^ in female in patients with a BMI ≥ 25 kg/m^2^; the X-tile program was applied to determine the optimal cut-off point for TAI (33.6 cm^2^/m^2^), SAI (23.3 cm^2^/m^2^), and VAI (30.6 cm^2^/m^2^).	8
Uojima et al. 2023	Japan	01/2019–04/2022	R	119	72 (37–83)^b^	98/21	Atezolizumab plus bevacizumab	SMI, SAI, VAI [OS(U), PFS(U)]	CT, L3, SliceOmatic	Median value (SMI = 42.2 cm^2^/m^2^, SAI = 40.3 cm^2^/m^2^, VFI = 43.2 cm^2^/m^2^)	7
Chen et al. 2023	China	08/2015–09/2021	R	111	59.0 ± 13.0^a^	97/14	Anti-PD-(L)1 antibody and/or anti-CTLA-4 antibody	SMI [OS(M), PFS(U), ORR(U), DCR(U)], SMD [OS(M), PFS(M)]	CT, L3, 3DSlicer	SMI, <40.8 cm^2^/m^2^ in males and <34.9 cm^2^/m^2^ in females; SMD, <41 HU in patients with a BMI < 25 kg/m^2^, and <33 HU in patients with a BMI ≥ 25 kg/m^2^	8
Chihiro et al. 2023	Japan	04/2018–04/2022	R	47	74 (55–90)^b^	36/11	Atezolizumab plus bevacizumab	SMI [OS(M), PFS(M)]	BIA, Inbody 720^®^/770^®^	1.86 kg/m^2^ in males and 5.7 kg/m^2^ in females	7
Oura et al. 2023	Japan	10/2020–01/2023	R	64	80 (67–91)^b^/72 (42–86)^b1^	49/15	Atezolizumab plus bevacizumab	SMI [OS(U)]	BIA, Inbody 770	7.0 kg/m^2^ for males and 5.7 kg/m^2^ for females	6
Guo et al. 2022	China	03/2020–12/2021	R	97	53.6 ± 10.7^a^/51.2 ± 11.0^a1^	7918	Camrelizumab	SMI [OS(U), PFS(M), ORR(U), DCR(U)]	CT/MRI, L3, Phillips Intelli Space Portal workstation	<37.7 cm^2^/m^2^ in males and <34.3 cm^2^/m^2^ in females	7
Toshida et al. 2022	Japan	04/2018–03/2022	R	35	74 (55–88)^b^/70 (36–4)^b1^	28/7	Atezolizumab plus bevacizumab	SMI [OS(U), PFS(U), ORR(U)]	CT, L3	<42 cm^2^/m^2^ in males and <38 cm^2^/m^2^ in females	6
Xiao et al. 2022	China	06/2018–10/2020	R	172	51.4 ± 11.7^a^	149/23	Nivolumab, Pembrolizumab, Sintilimab, Tislelizumab, Atezolizumab, Durvalumab, Avelumab	SMI, SMD, SAI, VAI, TAI [OS(M), PFS(M)]	CT, L3 SliceOmatic	SMI, <43 cm^2^/m^2^ in male and <41 cm^2^/m^2^ in female in patients with a BMI < 25 kg/m^2^, <53 cm^2^/m^2^ in male and <41 cm^2^/m^2^ in female in patients with a BMI ≥ 25 kg/m^2^; SMD, <41 HU in patients with a BMI < 24.9 kg/m^2^, and <33 HU in patients with a BMI > 25 kg/m^2^; the Youden Index was used to identify the optimal cut-off values for VAI, SAI, and TAI	7
Zhao et al. 2022	China	01/2018–12/2020	R	160	58 (26–86)^b^	129/31	Pembrolizumab, Nivolumab, Sintilimab, Camrelizumab	PMI[OS(M), PFS(M), ORR(U), DCR(U)]	CT, L3	Optimal cutoff (14.19)	7
Akce et al. 2021	China	2015–2019	R	57	66^d^	44/13	Anti-PD1 antibody	SMI [OS(M), PFS(M)]	CT/MRI, L3, SliceOmatic	<43 cm^2^/m^2^ in males and <39 cm^2^/m^2^ in females	6
Yang et al. 2021	China	01/2019–12/2022	R	96	57.04 ± 9.96	87/9	Toripalimab, Camrelizumab	SMI [OS(M)]	CT, L3, Core Slicer	<45.37 cm^2^/m^2^ in males and <36.33 cm^2^/m^2^ in females	7
Kim et al. 2021	Korea	03/2017–12/2018	R	102	61 (54–69)^c^	87/15	Nivolumab	SMI [OS(M), PFS(U), ORR(U), DCR(U)]	CT, L3, MATLAB version R2014a	<42 cm^2^/m^2^ in males and <38 cm^2^/m^2^ in female	7

R: retrospective study; M: multivariate analysis; U: univariate analysis; HCC: hepatocellular carcinoma; ICI: immune checkpoint inhibitor; PD-1: programmed cell death protein 1; PD-L1: programmed death-ligand 1; CTLA-4: cytotoxic T-lymphocyte-associated protein 4; SMI: skeletal muscle index; SMD: skeletal muscle density; PMI: psoas muscle index; SAI: subcutaneous adipose index; VAI: visceral adipose index; TAI: total adipose index; OS: overall survival; PFS: progression-free survival; ORR: objective response rate; DCR: disease control rate; MRI: magnetic resonance images; CT: computed tomography; L3: third lumbar vertebrae; MRI: magnetic resonance images; HU: Hounsfield units; BMI: body mass index.

^a^Mean ± standard deviation.

^b^Medians with ranges.

^c^Median and interquartile range.

^d^Medians.

^1^low SMI *vs.* high SMI.

### 3.3. Baseline skeletal muscle index (SMI) levels and overall survival and progression-free survival

**Figure 2.**
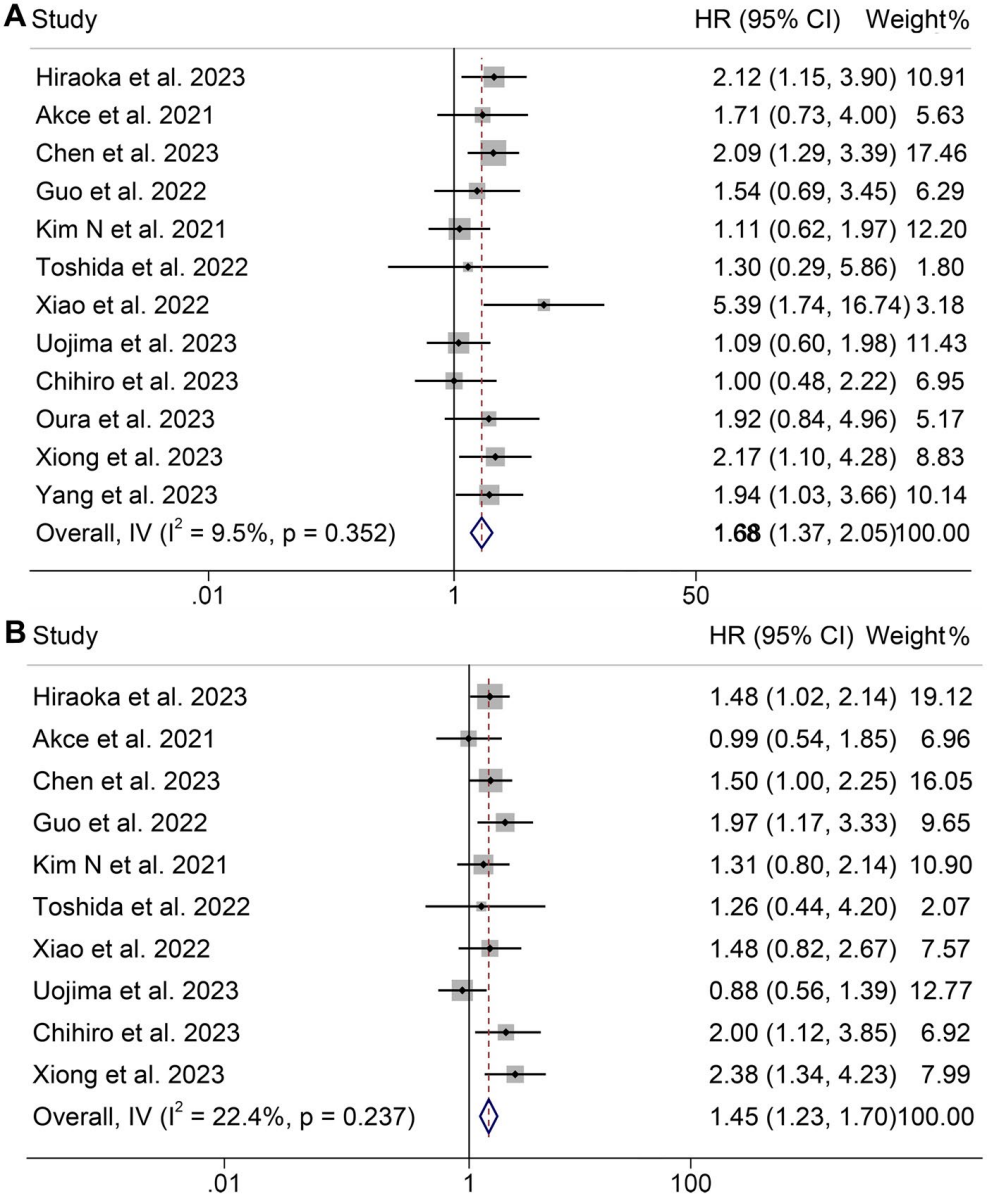
Forest plots of the relationship between skeletal muscle index and overall survival (A) and progression-free survival (B). HR: hazard ratio; CI: confidence interval. The results revealed that patients with low SMI had significantly inferior OS (HR: 1.68, 95% CI: 1.37–2.05, *p* < 0.001) and PFS (HR: 1.45, 95% CI: 1.23–1.70, *p* < 0.001) compared to those with high SMI.

A total of 12 studies involving 1203 patients were included in this analysis, examining the impact of pre-treatment SMI levels on OS or PFS in ICI-treated HCC patients. Our findings demonstrated that patients with low SMI had significantly inferior OS (HR: 1.68, 95% CI: 1.37–2.05, *p* < 0.001, Figure 2A) and PFS (HR: 1.45, 95% CI: 1.23–1.70, *p* < 0.001, Figure 2B) compared to those with high SMI. The Cochran Q test and I^2^ statistics indicated no significant heterogeneity (OS: *I*^2^ = 9.5%, *p* = 0.352; PFS: *I*^2^ = 22.4%, *p* = 0.237), indicating methodological homogeneity between studies; thus, a fixed-effects model was utilized.

### 3.4. Baseline skeletal muscle index (SMI) levels and immunotherapy responses

**Figure 3.**
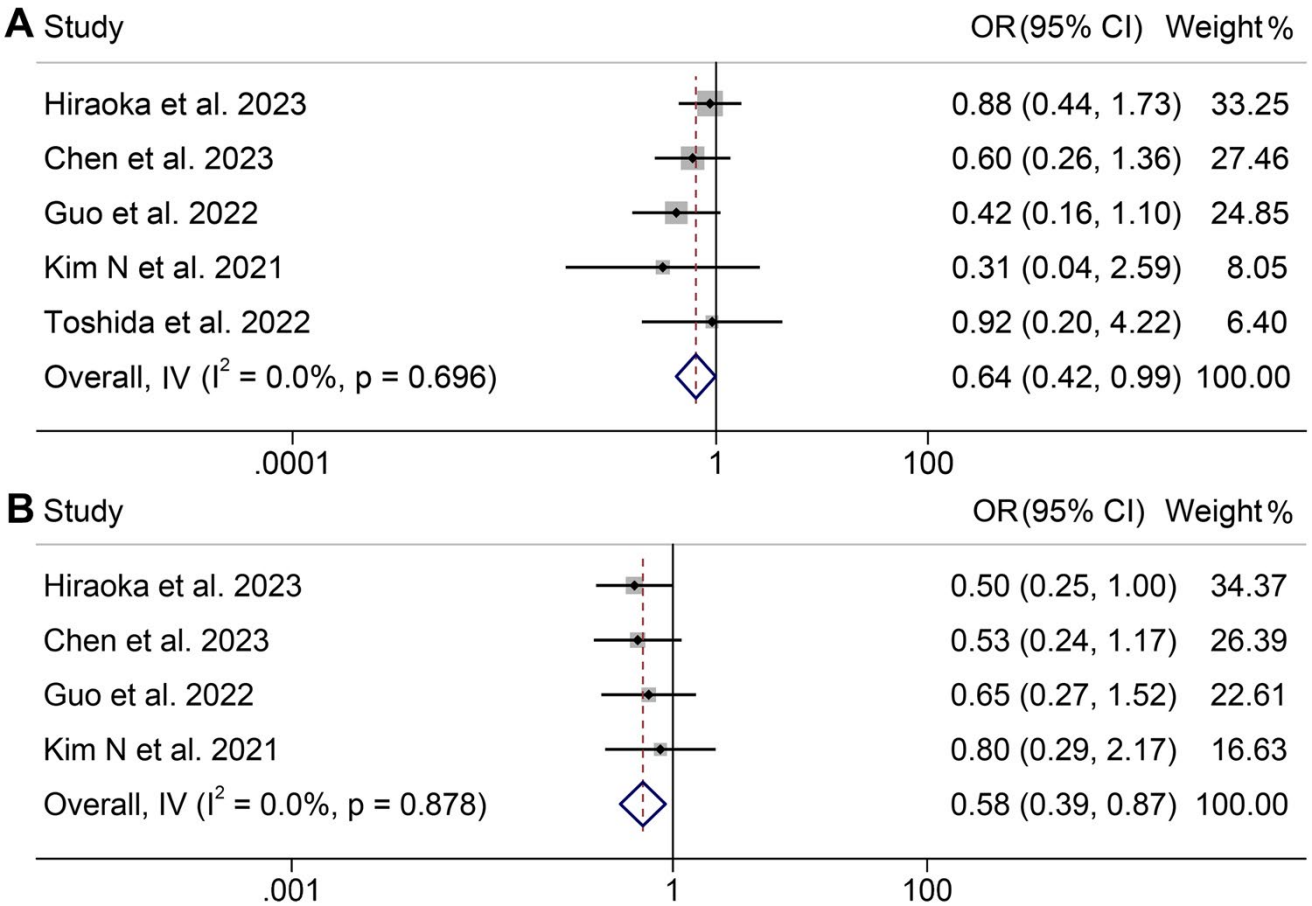
Forest plots of the relationship between skeletal muscle index and objective response rate (A) and disease control rate (B). OR: odds ratio; CI: confidence interval. The results revealed that low SMI patients exhibited a lower ORR (OR: 0.64, 95% CI: 0.42–0.99, *p* = 0.044) and DCR (OR: 0.58, 95% CI: 0.39–0.87, *p* = 0.009) compared to high SMI patients.

We analyzed the relationship between SMI levels and the ORR and DCR of HCC patients using five studies (574 patients) and four studies (539 patients), respectively. Notably, there was no significant heterogeneity among the included studies (ORR: *I*^2^ = 0, *p* = 0.696; DCR: *I*^2^ = 0, *p* = 0.878), thus a fixed-effects model was employed. The results demonstrated that low SMI patients exhibited a lower ORR (OR: 0.64, 95% CI: 0.42–0.99, *p* = 0.044, Figure 3A) and DCR (OR: 0.58, 95% CI: 0.39–0.87, *p* = 0.009, Figure 3B) compared to high SMI patients.

### 3.5. Pre-immunotherapy myosteatosis and overall survival and progression-free survival

**Figure 4.**
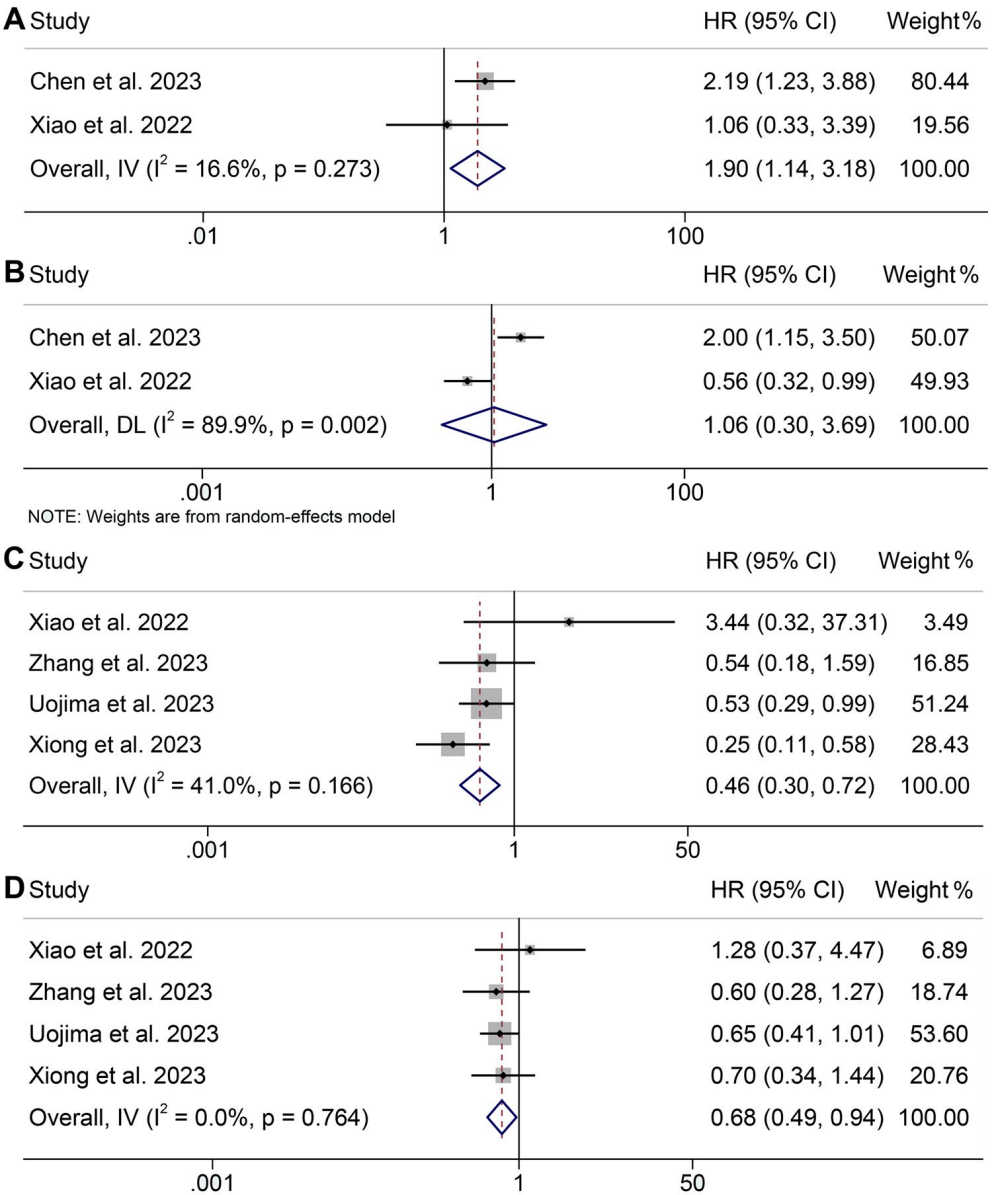
Forest plots of the relationship between myosteatosis and overall survival (A) and progression-free survival (B). Forest plots of the relationship between subcutaneous adipose index and overall survival (C) and progression-free survival (D). HR: hazard ratio; CI: confidence interval. The results revealed that HCC patients with myosteatosis had a significantly shorter OS (*I*^2^ = 16.6%, *p* = 0.273, HR: 1.90, 95% CI: 1.14–3.18, *p* = 0.015). The analysis showed no significant difference in PFS between patients with myosteatosis and those without myosteatosis (*I*^2^ = 89.9%, *p* = 0.002, HR: 1.06, 95% CI: 0.30–3.69, *p* = 0.928).

Two studies, including 283 patients, assessed the relationship between myosteatosis and OS and PFS in HCC patients. The pooled analysis revealed that HCC patients with myosteatosis had a significantly shorter OS (*I*^2^ = 16.6%, *p* = 0.273, HR: 1.90, 95% CI: 1.14–3.18, *p* = 0.015, Figure 4A). However, the analysis showed no significant difference in PFS between patients with myosteatosis and those without myosteatosis (*I*^2^ = 89.9%, *p* = 0.002, HR: 1.06, 95% CI: 0.30–3.69, *p* = 0.928, Figure 4B).

### 3.6. Baseline subcutaneous adipose index (SAI) and overall survival and progression-free survival

There were four studies with 421 patients reporting the correlation between SAI levels and OS and PFS in HCC patients treated with ICI immunotherapy. There was no significant heterogeneity among studies (OS: *I*^2^ = 41.0%, *p* = 0.166; PFS: *I*^2^ = 0, *p* = 0.764), and thus a fixed-effect model was used. The pooled result revealed that high SAI levels were associated with better OS (HR: 0.46, 95% CI: 0.30–0.72, *p* = 0.001, Figure 4C) or PFS (HR: 0.68, 95% CI: 0.49–0.94, *p* = 0.021, Figure 4D) than those with low SAI levels.

### 3.7. Pre-immunotherapy visceral adipose index (VAI), total adipose index (TAI), and overall survival and progression-free survival

Three studies (365 patients) and two studies (246 patients) examined the predictive roles of VAI and TAI on HCC patient prognosis, respectively. We found no correlation between the levels of VAI (OS, *I*^2^ = 80.5%, *p* = 0.006, HR: 0.47, 95% CI: 0.12–1.88, *p* = 0.288, Figure S1A; PFS, *I*^2^ = 0, *p* = 0.865, HR: 0.90, 95% CI: 0.62–1.32, *p* = 0.601, Figure S1B) and TAI (OS, *I*^2^ = 0, *p* = 0.377, HR: 0.52, 95% CI: 0.21–1.32, *p* = 0.183, Figure S1C; PFS, *I*^2^ = 0, *p* = 0.513, HR: 0.50, 95% CI: 0.24–1.03, *p* = 0.098, Figure S1D) and the prognosis of ICI-treated HCC patients.

### 3.8. Pre-immunotherapy sarcopenia and overall survival and progression-free survival

Twelve studies (1203 patients) used SMI, and two studies used PMI (284 patients) to diagnose sarcopenia. We, therefore, analyzed the relationship between sarcopenia and the prognosis of HCC patients treated with ICIs. The results of our analysis demonstrated that patients with sarcopenia had significantly shorter OS compared to those without sarcopenia (HR: 1.63, 95% CI: 1.29–2.04, *p* < 0.001, Figure S2A). Assessment of heterogeneity using the Cochran Q test and I^2^ statistics revealed significant heterogeneity among the studies (*p* = 0.020, *I*^2^ = 49.1%). Consequently, a random-effect model was employed to estimate the pooled effect.

We also investigated the relationship between sarcopenia and PFS in HCC patients treated with ICIs. Our analysis, depicted in Figure S2B, showed no significant heterogeneity among the studies (*I*^2^ = 17.9%, *p* = 0.274), allowing us to employ a fixed-effect model. The results revealed a significant association between sarcopenia and poorer PFS (HR: 1.48, 95% CI: 1.27–1.72, *p* < 0.001).

### 3.9. Sarcopenia and immunotherapy responses

A total of six articles, encompassing 734 patients, investigated the relationship between sarcopenia and ORR in HCC patients treated with ICIs. No significant heterogeneity was observed among the included studies (*I*^2^ = 0, *p* = 0.815), thus a fixed-effects model was employed. Our analysis revealed that sarcopenia patients had a lower ORR compared to non-sarcopenia patients (OR: 0.64, 95% CI: 0.44–0.93, *p* = 0.020, Figure S3A).

Furthermore, we analyzed data from five studies involving 699 individuals to examine the relationship between sarcopenia and DCR in HCC patients. No significant heterogeneity was found in these studies (*I*^2^ = 0, *p* = 0.954), and a fixed-effects model was utilized. Our findings indicated that HCC patients with sarcopenia had a lower DCR (OR: 0.58, 95% CI: 0.39–0.86, *p* = 0.007, Figure S3B) compared to those without sarcopenia.

### 3.10. Subgroup analyses

Compared with univariate Cox regression analysis, multivariate analysis can control for confounding factors, improve statistical power, and reduce estimation bias, providing a more reliable basis for clinical decision-making. Therefore, we performed subgroup analyses according to the Cox regression model. In the multivariate analysis, we found that low SMI and sarcopenia remained significantly associated with worse OS (SMI, HR: 1.80, 95% CI: 1.43–2.27, *p <* 0.001, Table 2; sarcopenia, HR: 1.73, 95% CI: 1.30–2.30, *p* < 0.001, Table S1) and PFS (SMI, HR: 1.64, 95% CI: 1.32–2.02, *p* < 0.001, Table 2; sarcopenia, HR: 1.66, 95% CI: 1.37–2.01, *p* < 0.001, Table S1).

**Table 2.** Subgroup analysis of the association between baseline skeletal muscle index and the outcomes of immune checkpoint inhibitors for hepatocellular carcinoma.

Variable	Included studies	Test of association			Test of heterogeneity		
		HR	95%CI	*p*-Value	Modal	*I* ^2^	*p*-Value
Overall survival							
Cox regression analysis							
Multivariate analysis	8	1.80	1.43–2.27	*p* < 0.001	F	26.7%	*p* = 0.215
Univariate analysis	4	1.36	0.90–2.04	*p* = 0.140	F	0	*p* = 0.752
Testing methods of body compositions							
CT/MRI	10	1.74	1.40–2.15	*p* < 0.001	F	11.9%	*p* = 0.333
BIA	2	1.32	0.74–2.36	*p* = 0.347	F	15.9%	*p* = 0.276
Progression-free survival							
Cox regression analysis							
Multivariate analysis	6	1.64	1.32–2.02	*p* < 0.001	F	8.7%	*p* = 0.361
Univariate analysis	4	1.22	0.95–1.57	*p* = 0.121	F	2.1%	*p* = 0.382
Testing methods of body compositions							
CT/MRI	9	1.41	1.19–1.67	*p* < 0.001	F	23.5%	*p* = 0.234
BIA	1	2.00	1.08–3.71	*p* = 0.028	–	–	–

BIA: bioelectrical impedance analysis; CT: computed tomography; MRI: magnetic resonance images; HR: hazard ratio; CL: confidence interval; F: fixed-effect model.

The subgroup analyses for the effect of the testing methods on body compositions for OS showed significant differences between the subgroups. Low SMI or sarcopenia was significantly associated with worse OS when using CT or MRI to detect body composition, whereas the above findings did not hold when using BIA (Table 2 and Table S1). The subgroup analyses on the influence of testing methods on body compositions for PFS revealed no significant differences between the subgroups (Table 2 and Table S1). In addition, sarcopenia, as diagnosed by PMI, was not significantly associated with shorter OS (Table S1).

### 3.11. Publication bias and sensitivity analysis

**Figure 5.**
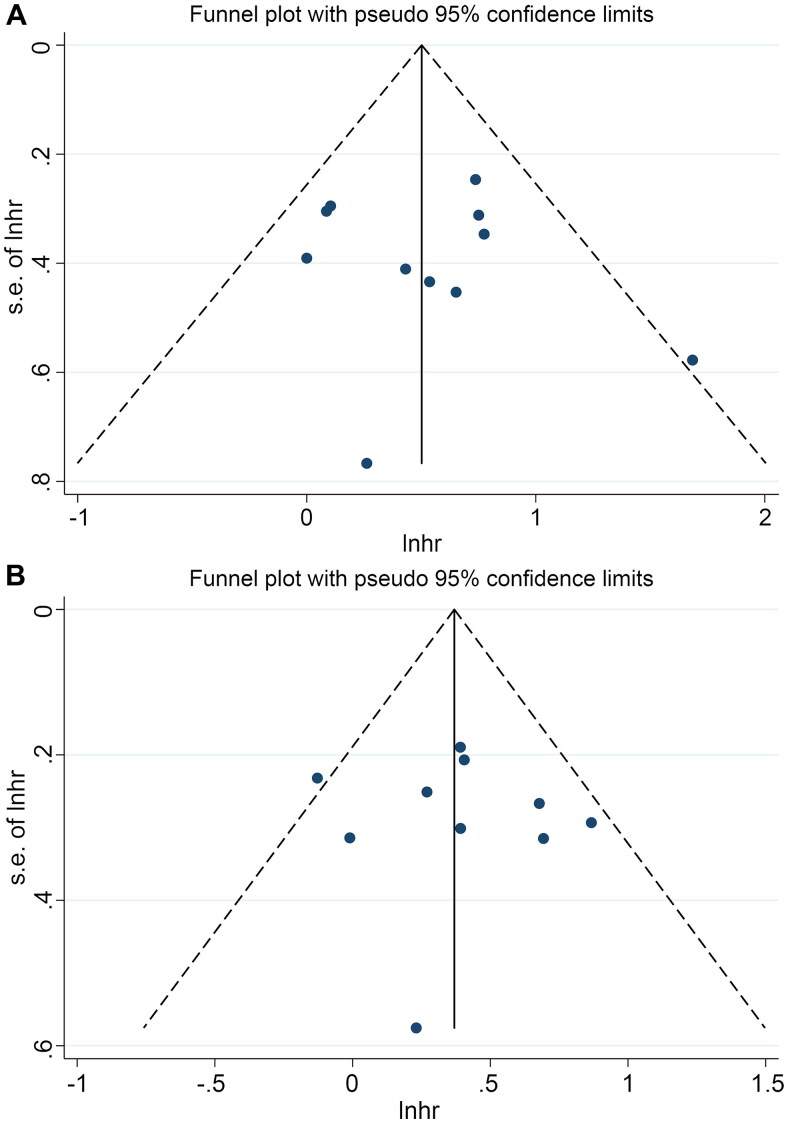
Funnel plots of the relationship between skeletal muscle index and overall survival (A) and progression-free survival (B). HR: hazard ratio. Funnel plots did not reveal significant publication bias in OS and PFS.

**Figure 6.**
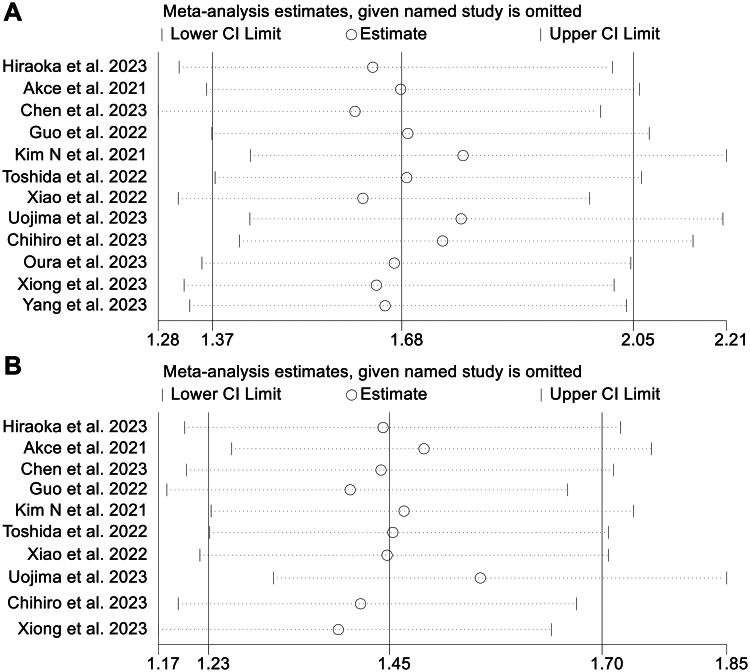
Sensitivity analysis of the association between skeletal muscle index and overall survival (A) and progression-free survival (B). HR: hazard ratio; CI: confidence interval. Funnel plots, Begg’s test, and Egger’s test did not reveal significant publication bias in OS (Egger’s test: *p* = 0.634, Begg’s test: *p* = 0.945, Figure 5(A)) and PFS.

SMI was used as the main indicator in this study, and we performed publication bias tests and sensitivity analysis on the results. Funnel plots, Begg’s test, and Egger’s test did not reveal significant publication bias in OS (Egger’s test: *p* = 0.634, Begg’s test: *p* = 0.945, Figure 5A) and PFS (Egger’s test: *p* = 0.790, Begg’s test: *p* = 1.000, Figure 5B). The sensitivity analysis, in which each study was excluded one at a time, demonstrated that the pooled HR for OS and PFS remained stable and robust (Figures 6A,B).

In addition, funnel plots and Egger’s test revealed a significant publication bias in the pooled analyses of the relationship between sarcopenia and OS (Egger’s test: *p* = 0.010, Begg’s test: *p* = 0.661, Figure S4A), whereas there was no significant publication bias in PFS (Egger’s test: *p* = 0.808, Begg’s test: *p* = 0.876, Figure S4B). Next, the number of missing studies in OS was calculated using the trim-and-fill method. After taking the missing hypothetical studies into account, the combined HR for OS was recalculated, but there were no substantial differences. Sensitivity analyses confirmed that these results were stable (Figures S5A,B).

## 4. Discussion

In this study, we found evidence from 13 studies that low SMI and sarcopenia were significantly related to worse OS and PFS, as well as lower ORR and DCR in HCC patients treated with ICIs. Furthermore, pooled results also revealed that high SAI might be significantly correlated with better PFS and OS in HCC patients. Conversely, we did not observe a significant association between myosteatosis, VAI, TAI, and survival in HCC patients receiving ICIs.

Sarcopenia is a muscular disorder characterized by a reduction in both muscle quantity and quality [36]. Detecting and diagnosing sarcopenia involves the use of various diagnostic assessments and methodologies. These encompass the application of the SARCF questionnaire, muscle strength measurements, skeletal muscle quantifications, physical performance evaluations, and anthropometric assessments [37]. Notably, within the domain of oncological research, muscle measurements conducted through CT scans, dual-energy X-ray absorptiometry (DXA), or bioelectrical impedance analysis (BIA) are commonly employed. However, the requirement of specialized equipment for DXA and the susceptibility of BIA accuracy to dehydration, a prevalent issue among cancer patients, pose certain limitations. In contrast, CT scans for tumor assessment are routinely administered to cancer patients. Consequently, within the oncology field, CT stands out as the preferred modality for sarcopenia diagnosis. SMI, calculated by dividing the total skeletal muscle area at the third lumbar vertebral level by the square of the patient’s height, serves as the most frequently utilized index in pertinent literature. It has demonstrated a strong correlation with overall body musculature and is associated with various health consequences [38]. Our study is consistent with the above findings that low SMI and sarcopenia detection by BIA are not significantly correlated with OS in HCC patients.

According to the European consensus statement, decreased muscle quantity or quality is essential for the diagnosis of sarcopenia [36]. The muscle mass area on CT imaging depicts muscle amount, whereas the muscle density reflects muscular quality. Muscle quality deterioration and fat infiltration into skeletal muscle can be indicators of muscular density loss. SMD is a frequently used metric for muscle quality that has been demonstrated to be a cancer prognosticator [39]. The current analysis, however, found that low SMD, i.e. myosteatosis, could not predict survival in HCC patients treated with ICIs.

It might be argued that low SMI or sarcopenia reflects a person’s advanced disease status and decreased physical condition, which results in a lower chance of survival. Our ORR and DCR data, however, imply that low SMI or sarcopenia is not only a prognostic factor, but also a predictive factor. Myokines, which are cytokines released by skeletal muscle, exert their effects through the autocrine, paracrine, and endocrine pathways [40]. Interleukin (IL)-15 is one of the myokines that enhances the proportion of circulating natural killer cells and CD8^+^ T cells [41]. Moreover, the administration of IL-15 in conjunction with ICIs increased the survival rate of mice with tumors [42]. Consequently, alterations in myokine levels due to low SMI or sarcopenia may impact the efficacy of ICI treatment. Contrary to visceral adipose tissue, subcutaneous fat serves as a physiological buffer for excessive energy intake and a useful lipid storage organ [43,44]. Subcutaneous fat tissue can also produce leptin, which favorably inhibits insulin activity [45], whereas insulin and insulin-like growth factors together represent a growth factor that promotes the invasion and growth of tumors [46].

Our conclusion that low SMI and sarcopenia lead to worse OS and PFS and lower ORR and DCR and that SAI is related to better survival in ICI-treated HCC patients is well supported by these preclinical studies. Thus, it is crucial to consider the detrimental effects of sarcopenia and low SMI on the efficacy of ICI immunotherapy in clinical practice. Further investigations are warranted to explore whether managing sarcopenia can improve the effectiveness of ICI treatment and to elucidate the underlying mechanisms driving the observed association between sarcopenia and reduced ICI efficacy.

Certain limitations should be acknowledged in this analysis. Firstly, the number of studies focusing on myosteatosis, PMI, SAI, VAI, and TAI is limited, and further confirmation is required to establish their impact on the effectiveness of ICI treatment. Additionally, it is important to note that all of the research included in our analysis was from Asia, implying that the findings of our study may be valid only for Asian populations. Finally, the cut-off values for the same diagnostic metric differed among investigations. However, no significant heterogeneity was found in the analyses for either SMI or sarcopenia, suggesting homogeneity and reliability across studies.

## 5. Conclusion

The predictive effect of low SMI and sarcopenia on outcomes in ICI-treated HCC patients is highlighted by this analysis. This finding is in favor of considering sarcopenia status when determining the prognosis for this patient population.

## Supplementary Material

Supplemental Material

## Data Availability

The data that support the findings of this study are available from the corresponding author upon reasonable request.
