# Supplementary material for: Prognostic impact of body composition in hepatocellular carcinoma patients with immunotherapy
Source: Ann Med. 2024 Aug 27;56(1):2395062. doi: 10.1080/07853890.2024.2395062 (PMC11351359; doi:10.1080/07853890.2024.2395062)
Supplement: Supplemental Material [file IANN_A_2395062_SM4007.zip › Supp/Supplementary material 1.docx]

**Supplementary material 1.** Detailed search strategy

(((((((((((((((((((((((Skeletal Muscle Index) OR (SMI)) OR (Psoas Muscle Index)) OR (PMI)) OR (Subcutaneous Adipose Index)) OR (SAI)) OR (Subcutaneous Fat Index)) OR (SFI)) OR (Visceral Adipose Index)) OR (VAI)) OR (Visceral Fat Index)) OR (VFI)) OR (Intramuscular Adipose Index)) OR (Intramuscular Fat Index)) OR (Muscle Surface Area)) OR (MSA)) OR (Skeletal Muscle Density)) OR (SMD)) OR (Myosteatosis)) OR (Psoas Muscle Mass) OR (Skeletal Muscle Mass) OR (Sarcopenia)) OR (Sarcopenic)) OR (((Body Composition) OR (Body Compositions)) OR ("Body Composition"[Mesh]))) AND ((((((((((((((((((((((((((((((((((((((((((((((((Camrelizumab) OR (Sintilimab)) OR (Tislelizumab)) OR (Toripalimab)) OR (Envafolimab)) OR (Immune Checkpoint Inhibitors)) OR (Checkpoint Inhibitors, Immune)) OR (Immune Checkpoint Inhibitor)) OR (Checkpoint Inhibitor, Immune)) OR (Immune Checkpoint Blockers)) OR (Checkpoint Blockers, Immune)) OR (Immune Checkpoint Blockade)) OR (Checkpoint Blockade, Immune)) OR (Immune Checkpoint Inhibition)) OR (Checkpoint Inhibition, Immune)) OR (PD-L1 Inhibitors)) OR (PD L1 Inhibitors)) OR (PD-L1 Inhibitor)) OR (PD L1 Inhibitor)) OR (Programmed Death-Ligand 1 Inhibitors)) OR (Programmed Death Ligand 1 Inhibitors)) OR (PD-1-PD-L1 Blockade)) OR (Blockade, PD-1-PD-L1)) OR (PD 1 PD L1 Blockade)) OR (CTLA-4 Inhibitors)) OR (CTLA 4 Inhibitors)) OR (CTLA-4 Inhibitor)) OR (CTLA 4 Inhibitor)) OR (Cytotoxic T-Lymphocyte-Associated Protein 4 Inhibitors)) OR (Cytotoxic T Lymphocyte Associated Protein 4 Inhibitors)) OR (Cytotoxic T-Lymphocyte-Associated Protein 4 Inhibitor)) OR (Cytotoxic T Lymphocyte Associated Protein 4 Inhibitor)) OR (PD-1 Inhibitors)) OR (PD-1 Inhibitor)) OR (PD 1 Inhibitors)) OR (Inhibitor, PD-1)) OR (PD 1 Inhibitor)) OR (Programmed Cell Death Protein 1 Inhibitor)) OR (Programmed Cell Death Protein 1 Inhibitors)) OR (Pembrolizumab)) OR (Nivolumab)) OR (Atezolizumab)) OR (Ipilimumab)) OR (Avelumab)) OR (Tremelimumab)) OR (Durvalumab)) OR (Cemiplimab)) OR (Immune Checkpoint Inhibitors[MeSH Terms])))
