# Supplementary material for: Prognostic impact of body composition in hepatocellular carcinoma patients with immunotherapy
Source: Ann Med. 2024 Aug 27;56(1):2395062. doi: 10.1080/07853890.2024.2395062 (PMC11351359; doi:10.1080/07853890.2024.2395062)
Supplement: Supplemental Material [file IANN_A_2395062_SM4007.zip › Supp/Supplementary materials Captions.docx]

**Supplementary materials Captions**

**Supplementary material 1**. Detailed search strategy.

**Table S1**. Subgroup analysis of the association between sarcopenia and the outcomes of immune checkpoint inhibitors for hepatocellular carcinoma.

**Figure S1**. Forest plots of the relationship between visceral adipose index and overall survival (A) and progression-free survival (B). Forest plots of the relationship between total adipose index and overall survival (C) and progression-free survival (D). HR, hazard ratio; CI, confidence interval.

**Figure S2**. Forest plots of the relationship between sarcopenia and overall survival (A) and progression-free survival (B). HR, hazard ratio; CI, confidence interval.

**Figure S3**. Forest plots of the relationship between sarcopenia and objective response rate (A) and disease control rate (B). OR, odds ratio; CI, confidence interval.

**Figure S4**. Funnel plots of the relationship between sarcopenia and overall survival (A) and progression-free survival (B). HR, hazard ratio

**Figure S5.** Sensitivity analysis of the association between sarcopenia and overall survival (A) and progression-free survival (B). HR, hazard ratio; CI, confidence interval.
