# Supplementary material for: Prognostic impact of body composition in hepatocellular carcinoma patients with immunotherapy
Source: Ann Med. 2024 Aug 27;56(1):2395062. doi: 10.1080/07853890.2024.2395062 (PMC11351359; doi:10.1080/07853890.2024.2395062)
Supplement: Supplemental Material [file IANN_A_2395062_SM4007.zip › Supp/Table S1 (6).docx]

| **Table S1.** Subgroup analysis of the association between sarcopenia and the outcomes of immune checkpoint inhibitors for hepatocellular carcinoma | | | | | | | | |
| --- | --- | --- | --- | --- | --- | --- | --- | --- |
| Variable | Included studies | Test of association | | | | Test of heterogeneity | | |
|  |  | HR | 95%CI | *p* value |  | Modal | I^2^ | *p* value |
| **Overall survival** |  |  |  |  | |  |  |  |
| Cox regression analysis | |  |  |  | |  |  |  |
| Multivariate analysis | 10 | 1.73 | 1.30-2.30 | *p* < 0.001 | | R | 63.0% | *p* = 0.004 |
| Univariate analysis | 4 | 1.36 | 0.90-2.04 | *p* = 0.140 | | R | 0 | *p* = 0.752 |
| Testing methods of body compositions | | |  |  | |  |  |  |
| CT/MRI | 12 | 1.68 | 1.30-2.16 | *p* < 0.001 | | R | 54.8% | *p* = 0.011 |
| BIA | 2 | 1.33 | 0.71-2.51 | *p* = 0.377 | | R | 15.9% | *p* = 0.276 |
| Body compositions |  |  |  |  | |  |  |  |
| SMI | 12 | 1.68 | 1.36-2.08 | *p* < 0.001 | | R | 9.5% | *p =* 0.352 |
| PMI | 2 | 1.57 | 0.75-3.30 | *p* = 0.234 | | R | 81.5% | *p =* 0.020 |
| **Progression-free survival** | |  |  |  | |  |  |  |
| Cox regression analysis |  |  |  |  | |  |  |  |
| Multivariate analysis | 7 | 1.66 | 1.37-2.01 | *p* < 0.001 | | F | 0 | *p* = 0.475 |
| Univariate analysis | 4 | 1.22 | 0.95-1.57 | *p* = 0.121 | | F | 2.1% | *p* = 0.382 |
| Testing methods of body compositions | | |  |  | |  |  |  |
| CT/MRI | 10 | 1.45 | 1.24-1.70 | *p* < 0.001 | | F | 19.5% | *p* = 0.263 |
| BIA | 1 | 2.00 | 1.08-3.71 | *p* = 0.028 | | - | - | - |
| Body compositions |  |  |  |  | |  |  |  |
| SMI | 10 | 1.45 | 1.23-1.70 | *p* < 0.001 | | F | 22.4% | *p* = 0.237 |
| PMI | 1 | 1.76 | 1.09-2.84 | *p* = 0.020 | | - | - | - |
| BIA, bioelectrical impedance analysis; CT, computed tomography; MRI, magnetic resonance images; SMI, skeletal muscle index; PMI, psoas muscle index; HR, hazard ratio; CL, confidence interval; F, fixed-effect model; R, random-effect model. | | | | | | | | |
