# Supplementary figures and images for: Prognostic impact of body composition in hepatocellular carcinoma patients with immunotherapy
Source: Ann Med. 2024 Aug 27;56(1):2395062. doi: 10.1080/07853890.2024.2395062 (PMC11351359; doi:10.1080/07853890.2024.2395062)

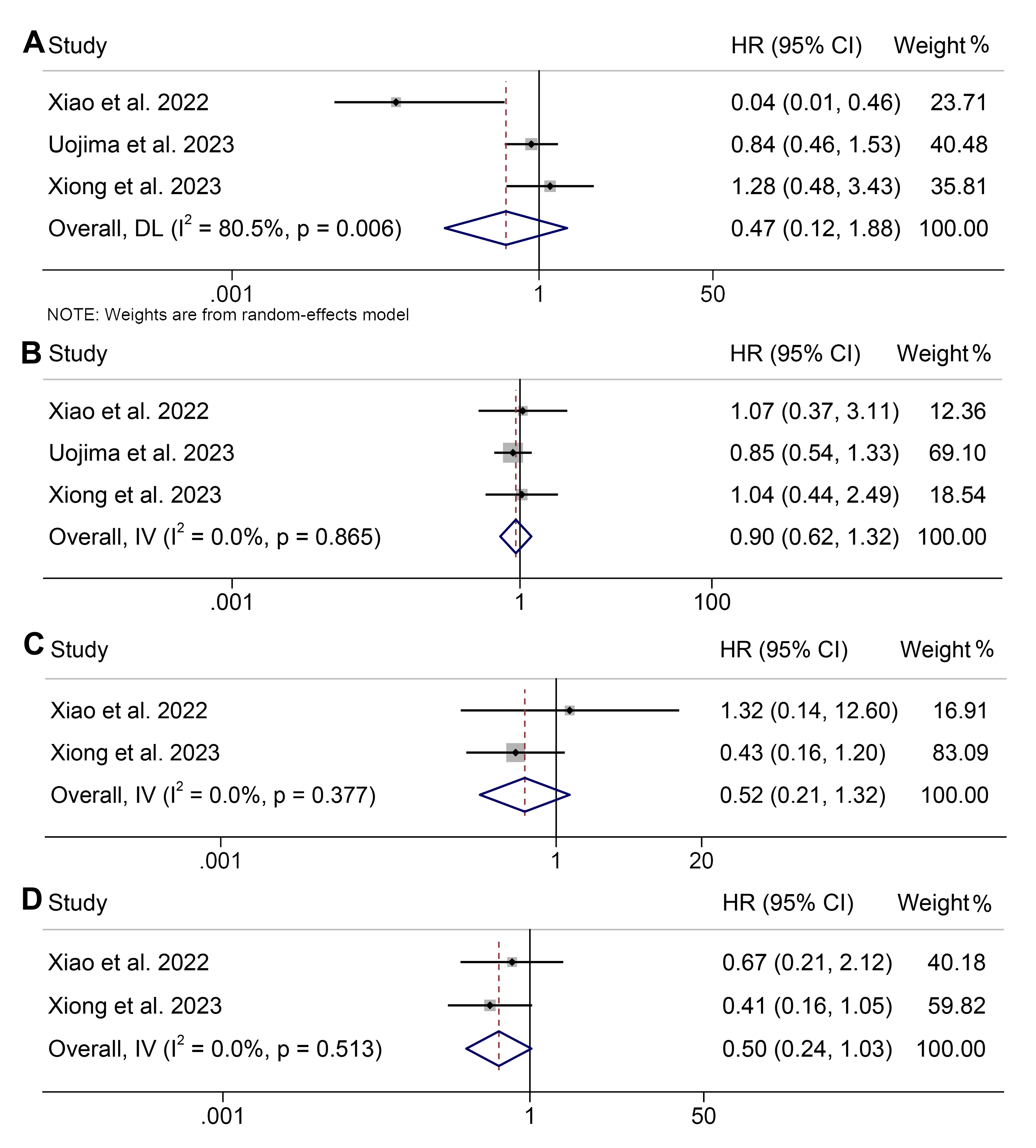

Supplement: Supplemental Material [file IANN_A_2395062_SM4007.zip › Supp/Figure S1 (3).tif]

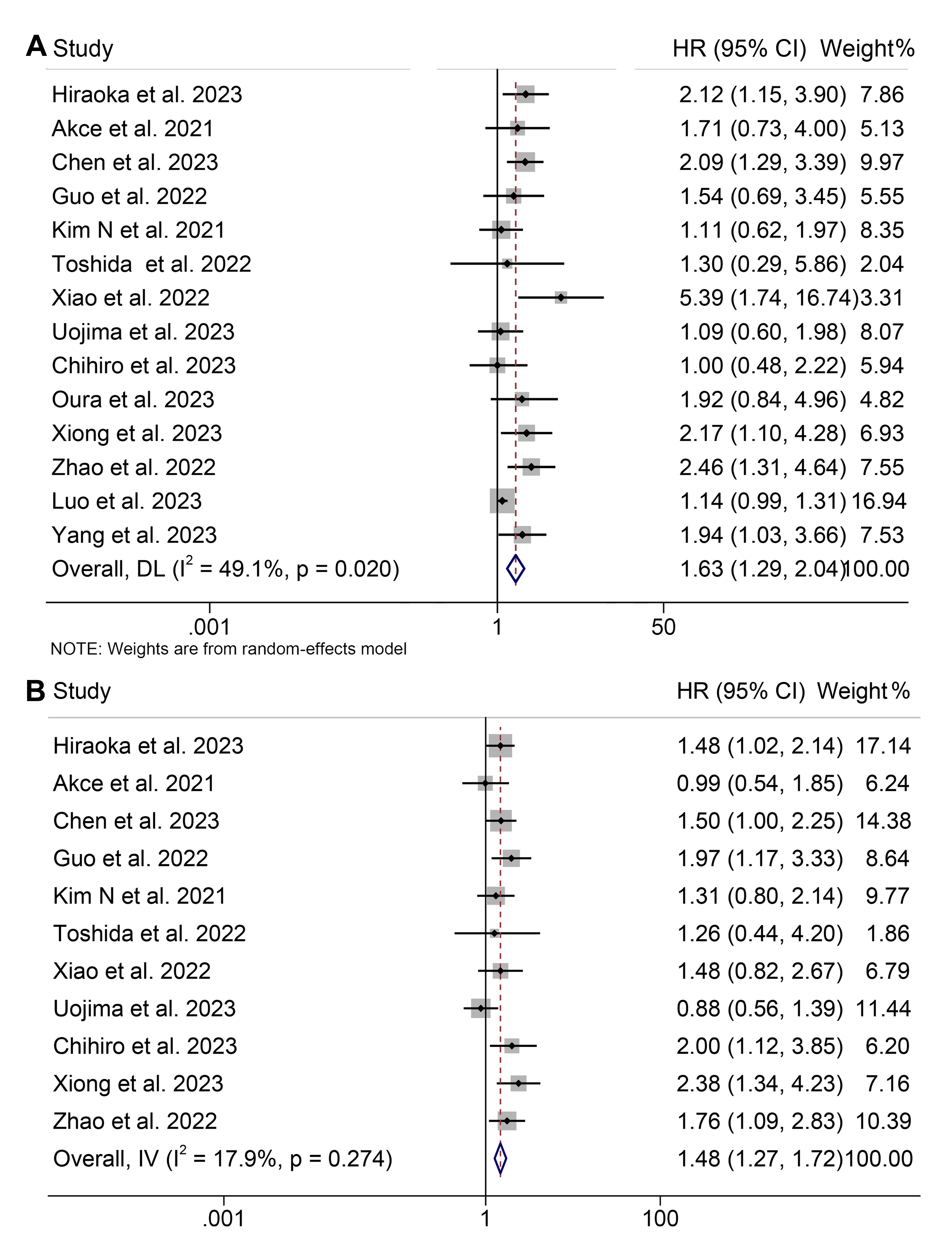

Supplement: Supplemental Material [file IANN_A_2395062_SM4007.zip › Supp/Figure S2.tif]

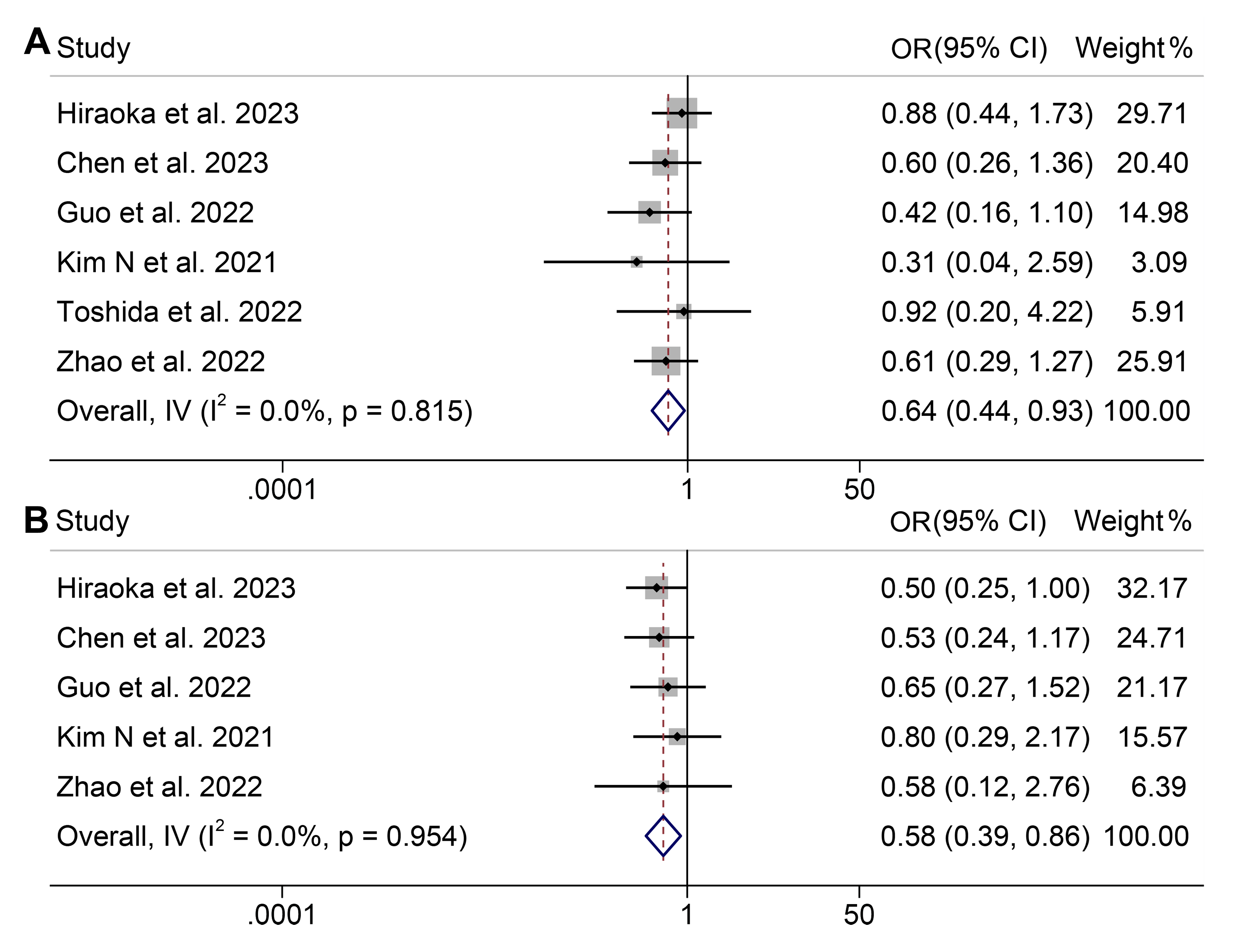

Supplement: Supplemental Material [file IANN_A_2395062_SM4007.zip › Supp/Figure S3.tif]

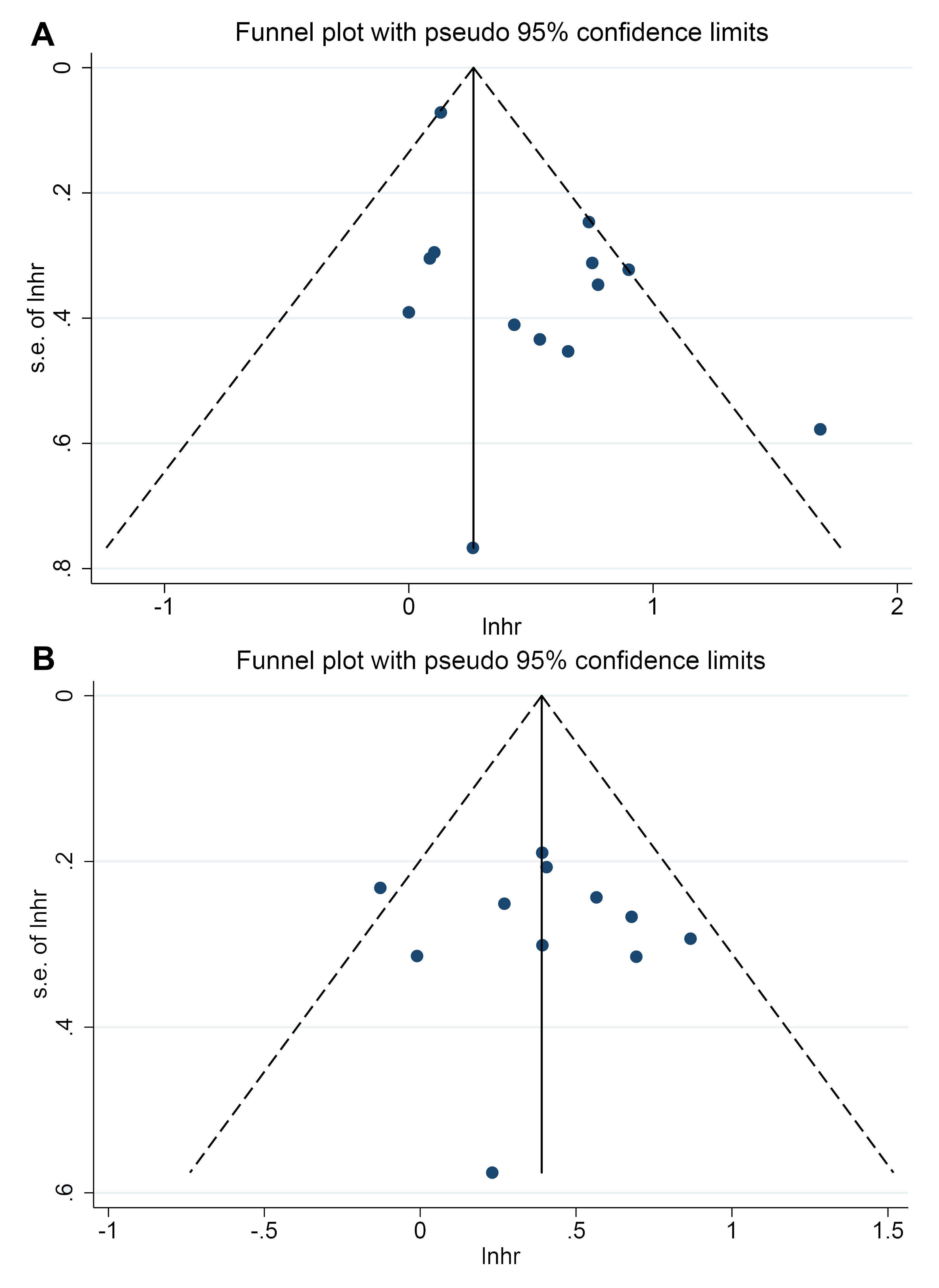

Supplement: Supplemental Material [file IANN_A_2395062_SM4007.zip › Supp/Figure S4.tif]

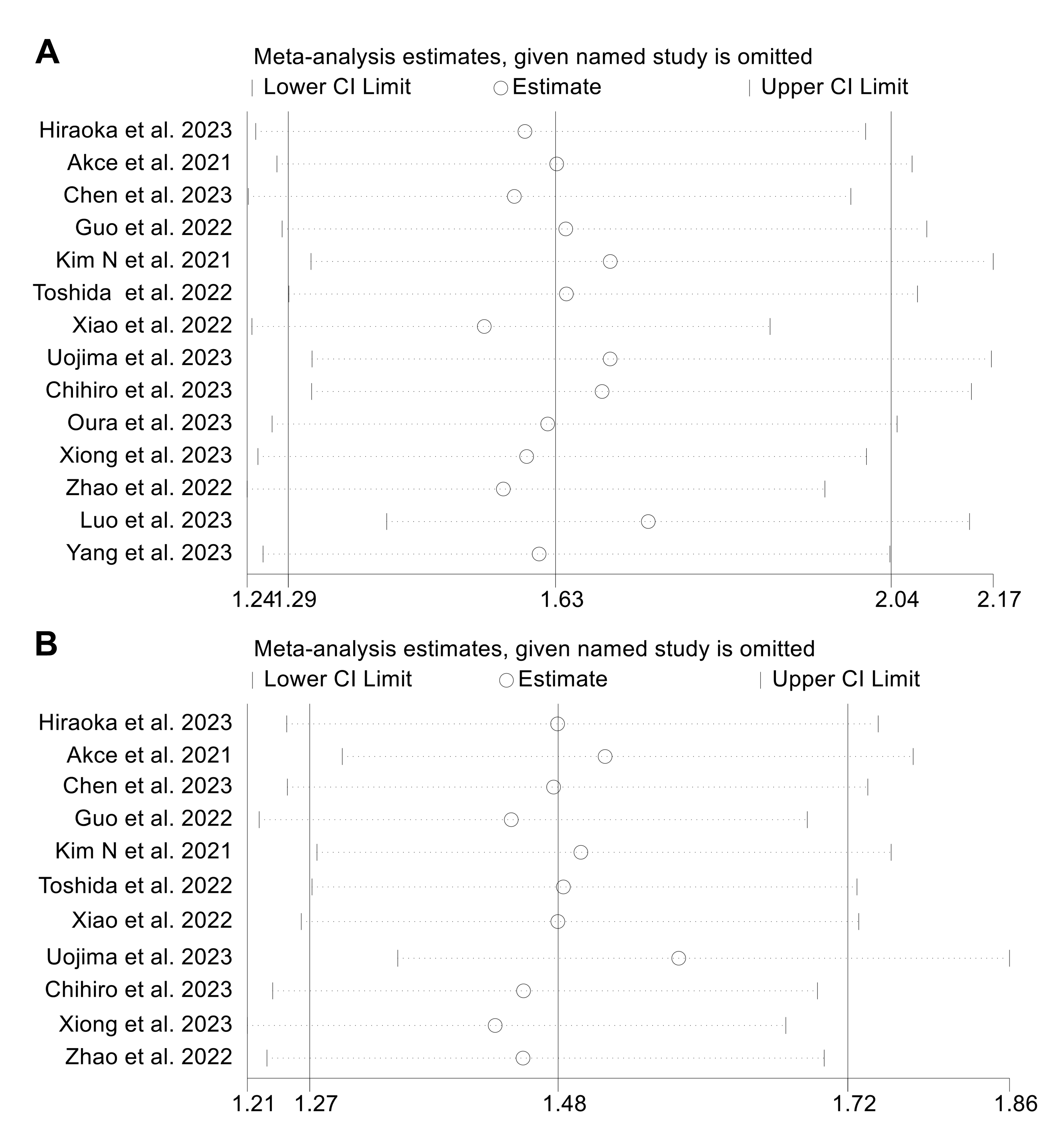

Supplement: Supplemental Material [file IANN_A_2395062_SM4007.zip › Supp/Figure S5.tif]
